# Considerations on the use of nucleic acid-based amplification for malaria parasite detection

**DOI:** 10.1186/1475-2875-10-323

**Published:** 2011-10-28

**Authors:** Stéphane Proux, Rossarin Suwanarusk, Marion Barends, Julien Zwang, Ric N Price, Mara Leimanis, Lily Kiricharoen, Natthapon Laochan, Bruce Russell, François Nosten, Georges Snounou

**Affiliations:** 1 Shoklo Malaria Research Unit, Mae Sot, Thailand; 2 Global Health Division, Menzies School of Health Research, Darwin, NT, Australia; 3 Singapore Immunology Network, A*STAR, Singapore; 4 Centre for Tropical Medicine, Nuffield Department of Clinical Medicine, University of Oxford, UK; 5 Muséum National d'Histoire Naturelle, Paris, France; 6 INSERM UMR S 945, F-75013 Paris, France; 7 Université Paris 6, Pierre & Marie Curie, Faculté de Médecine Pitié-Salpêtrière, F-75013 Paris, France

## Abstract

**Background:**

Nucleic acid amplification provides the most sensitive and accurate method to detect and identify pathogens. This is primarily useful for epidemiological investigations of malaria because the infections, often with two or more *Plasmodium *species present simultaneously, are frequently associated with microscopically sub-patent parasite levels and cryptic mixed infections. Numerous distinct equally adequate amplification-based protocols have been described, but it is unclear which to select for epidemiological surveys. Few comparative studies are available, and none that addresses the issue of inter-laboratory variability.

**Methods:**

Blood samples were collected from patients attending malaria clinics on the Thai-Myanmar border. Frozen aliquots from 413 samples were tested independently in two laboratories by nested PCR assay. Dried blood spots on filter papers from the same patients were also tested by the nested PCR assay in one laboratory and by a multiplex PCR assay in another. The aim was to determine which protocol best detected parasites below the sensitivity level of microscopic examination.

**Results:**

As expected PCR-based assays detected a substantial number of infected samples, or mixed infections, missed by microscopy (27 and 42 for the most sensitive assay, respectively). The protocol that was most effective at detecting these, in particular mixed infections, was a nested PCR assay with individual secondary reactions for each of the species initiated with a template directly purified from the blood sample. However, a lesser sensitivity in detection was observed when the same protocol was conducted in another laboratory, and this significantly altered the data obtained on the parasite species distribution.

**Conclusions:**

The sensitivity of a given PCR assay varies between laboratories. Although, the variations are relatively minor, they primarily diminish the ability to detect low-level and mixed infections and are sufficient to obviate the main rationale to use PCR assays rather than microscopy or rapid diagnostic tests. The optimal approach to standardise methodologies is to provide PCR template standards. These will help researchers in different settings to ensure that the nucleic acid amplification protocols they wish to use provide the requisite level of sensitivity, and will permit comparison between sites.

## Background

Microscopic examination of Giemsa-stained blood smears remains the most reliable method for routine clinical diagnosis of malaria. The technique is robust and except for minor variations, it has remained unmodified since the invention of the thick smear more than 108 years ago [1]. At present, reliable and prompt microscopic malaria diagnosis is sub-optimal in many endemic regions because material, training and transport costs restrict the availability of suitably equipped, experienced microscopists. There are now many different commercially available rapid diagnostic tests (RDTs), and those that equal the sensitivity and specificity of blood smear examination are poised to replace or supplement microscopy.

Parasite detection and accurate identification are equally important in epidemiological surveys, because the design, implementation and monitoring of control measures are directly based on these data. However, neither microscopy nor rapid diagnostic tests can ensure accurate estimates of parasite prevalence. First, the number of endemic residents with sub-microscopic infections often exceeds that of persons with microscopically patent parasitaemia. This is because the untreated malaria infection is predominantly a low-grade often sub-patent chronic infection that usually persists for many months [2]. Moreover, in the immune and semi-immune person newly acquired infections are likely to remain sub-clinical and sub-patent [3]. The improved sensitivity of detection afforded by thick smear microscopy is offset by poor parasite morphology, which makes species identification unreliable, particularly at low parasite levels. Currently, there are many rapid diagnostic tests that can specifically detect *Plasmodium falciparum *and *Plasmodium vivax*, but none that achieve this for all the *Plasmodium *species that infect humans http://www.wpro.who.int/sites/rdt/home.htm. Finally, neither microscopy nor rapid diagnostic tests are suited for the detection and identification of parasites in the mosquito.

The magnitude of the discrepancy between the data obtained from microscopic examination and true parasite prevalence was made evident when protocols based on nucleic acid amplification were introduced. One of the first protocols described achieved absolute sensitivity and specificity for the four species of *Plasmodium *that infect humans [4]. It was based on a nested polymerase chain reaction (PCR) amplification of the parasites' small subunit ribosomal RNA genes (ssrRNA), a target previously shown to be suitable for diagnostics [5]. It was evident from the first two sets of field samples subjected to this PCR assay [4,6,7] that microscopy alone significantly underestimated the prevalence of malaria infections, in particular missing most of the mixed species infections. These observations were confirmed in nearly all studies conducted subsequently. PCR amplification using standard thermal cyclers is now routinely available in many endemic settings, and its costs have decreased over the last years. Therefore, it has become possible to envisage its inclusion in epidemiological surveys.

There is now a plethora of PCR-based detection assays for *Plasmodium *parasites that infect humans, most of which are based on the ssrRNA genes. However, it is unclear which to select for routine surveys because extensive comparative studies are neither available nor likely to be undertaken. There are two basic approaches for species detection, nested PCR or single PCR. In general nested PCR is more sensitive than single PCR, but multiple reactions are needed in order to establish diagnosis. Generally, primers that would support amplification of the target irrespective of the *Plasmodium *species are used in the primary amplification, while species-specific oligos are used for the secondary amplification, either individually in separate reactions (up to six if the presence of both *Plasmodium ovale *types and *Plasmodium knowlesi *is also sought). Detection can be achieved by a single amplification reaction where all the oligos needed to detect the different species are multiplexed, either as a single PCR or as the secondary reaction of a nested PCR protocol. Multiplexing of oligonucleotides very often diminishes sensitivity, in particular for the minor population [8].

At present, our perception of malaria epidemiology is almost exclusively derived from microscopic examination of Giemsa-stained blood slides. The data, which are most often collected from passive case detection records/surveys, are used to formulate and monitor malaria control strategies. The ultimate aim of the present study was to determine whether PCR detection of *Plasmodium *provided a substantially improved picture of the presence and type of malaria parasites in the samples tested. Irrespective of the methodology used, the sensitivity of any PCR assay rests firmly on the quantity and the quality of the template used to initiate the reaction and on adequate optimization of the amplification protocols. In this article these issues were addressed by comparing the data obtained from two distinct PCR strategies, nested PCR [9] and single multiplexed PCR [10], independently applied to the same set of samples. Whether the data derived from the nested PCR protocol varied when the work was independently conducted in two laboratories, or within one laboratory using DNA templates that were prepared using different methods, was also addressed.

## Methods

### Sample collection

Samples were collected from febrile patients presenting at clinics of the Shoklo Malaria Research Unit (SMRU) and screened as part of treatment trials approved by the Oxford University Tropical Research Ethics committee (OXTREC) and the Faculty of Tropical Medicine Ethics Committee.

The blood was collected from a single finger prick made on the side of the third finger after disinfection of the area with cotton soaked in 70% alcohol. The first drop of blood was removed and 150 μl (children) or 250 μl (adults) of capillary blood were collected in Microtainer tubes supplemented with K_2 _EDTA (Beckton-Dickinson, ref 365974). The samples were then kept at room temperature and brought back to the laboratory in Mae-Sot within 12 hours. Upon reception, three aliquots were made for each sample; each composed of 25 μl of the blood mixed with 5 μl of 50 mM EDTA pH 8.0 (final EDTA concentration would be 18 mM or 24 mM depending on the original blood volume collected). One aliquot was shipped frozen to Paris, another aliquot was assayed by the team in Thailand, and the third kept as a back up. Approximately 30 μl of blood were spotted on Whatman 3 MM filter paper in triplicate. The filters were allowed to dry protected from direct sunlight and insects and then stored in individual sealable plastic bags containing desiccant. One set was assayed by the team in Thailand; the other was shipped to Australia where it was assayed; the third aliquot was kept as a back up.

### Microscopy

Blood smears were left to dry immediately after collection before staining with a 10% Giemsa solution with buffer water ph 7.2 for 20 minutes. Thin smears were fixed with absolute methanol prior to Giemsa staining. Smears were examined on an Olympus microscope at a magnification of × 1000. The blood smears were read at the time of collection by the clinics' microscopists. The same set of slides was then read blind within a couple of days by senior microscopists at SMRU. The discrepancies between the two readings were resolved by the senior microscopist (S. Proux). For the first reading, slides were declared negative when no parasites were found in 100 thick smear fields. For the second reading, slides were declared negative when no parasites were found in 200 thick smear fields. Parasitaemia was calculated from the number of parasites observed per 500 white blood cells, though when the count exceeded 500 parasites per 500 white blood cells, the count was obtained by enumeration of the parasites in a fixed number of thin smear fields (5, 10 or 20 fields depending on the parasite numbers). It was considered that there were 8,000 white blood cells per microlitre of blood, and that each thin smear field had 200 red blood cells. Calculation of the number of parasites per microlitre of blood was done as follows: number of parasites observed per 500 white blood cells in thick films × 16; number of parasites observed in 1,000 red blood cells in the thin smear x the haematocrit (considered to be 38% for this population) × 125.6. For the purposes of this study, it was not felt that a more accurate measure of low parasite burdens based on individual white blood cell counts [11] was warranted.

### PCR template preparation

The PCR analyses were carried out independently in three laboratories: the Shoklo Malaria Research Unit in Thailand (SMRU), the Muséum National d'Histoire Naturelle in Paris (MNHN), and at the Menzies School of Health Research in Australia (MSHR).

For the samples collected on filter paper, the template for the PCR assays was purified from all the blood spotted on the filter paper, approximately 30 μl. At SMRU, the template was prepared by Chelex extraction of a blood sample present on a piece of the filter of approximately 1 cm in diameter (ca. 25 μl o blood) to yield a template solution of 125 μl. At MSHR, the template was purified from each filter paper blood spot using the QIAamp^® ^DNA MiniKits, yielding a template solution of 100 μl.

For the uncoagulated blood samples, the template used for each PCR assay was semi-purified as follows. The frozen blood sample was thawed out on ice and a 5.0 μl aliquot was removed and placed in a tube containing 400 μl of PBS. After mixing, the tube was centrifuged at room temperature for 5 min at 10,000 × g. The supernatant was carefully removed by suction with a fine drawn-out glass pipette, and another 400 μl of PBS were added and the tube gently inverted a couple of times before being subjected to a second round of centrifugation as above. Once the supernatant was carefully removed, the reaction mixture for the primary PCR amplification was added directly to the pellet, and the tubes placed in the thermal cycler after overlaying the mixture with 50 μl of mineral oil. As a result of the procedure described above the levels of haemoglobin and EDTA, both potent inhibitors of PCR amplification, would have been reduced to minimal levels.

### PCR protocols

The nested PCR protocol (Nes) and primers were used as previously described [9], except that an additional oligonucleotide primer pair was used for the detection of variant *P. ovale *[12]. Briefly, in the primary reaction the template was amplified using primers that recognise the ssrRNA genes from all *Plasmodium *species (rPLU1 and rPLU5). One μl of the product obtained after 30 cycles was then used in four separate secondary reactions in which one or other of the four species-specific primer pairs were added. The primary amplification reaction was initiated using one μl of the template from the blood samples spotted on the filter paper (F), which corresponds to an aliquot of 0.2 μl or 0.3 μl of blood (chelex or Qiagen extraction, respectively), or directly from the uncoagulated blood sample (B), which corresponds to an aliquot of 5.0 μl of blood.

The multiplex PCR protocol (Mul) was used as previously described [10]. The template for these amplification reactions was purified from the blood sample dried on filter paper (F).

The PCR analyses were carried out independently in three laboratories: the Shoklo Malaria Research Unit in Thailand (SMRU), the Muséum National d'Histoire Naturelle in Paris (MNHN), and at the Menzies School of Health Research in Australia (MSHR). The different assays were coded as follows: the type of PCR assay-laboratory where it was carried out-the type of template employed (Nes or Mul - SMRU, MNHN or MSHR - B or F; respectively).

At the MNHN, the polymerase used was AmpliTaq^® ^(Applied Biosystems) and the thermocycler used was the PTC-100 (MJ Research); at SMRU the polymerase used was BIOTAQ™ DNA polymerase (Bioline) and the thermocycler used was the GeneAmp^® ^PCR System 9700 (Applied Biosystems); at MSHR the polymerase used was AmpliTaq^® ^(Applied Biosystems) and the thermocycler used was Corbett Research 96-well Gradient Palm-Cycler.

### Statistical analysis

Statistical analyses were carried out using the McNemar paired test to compare the difference between PCR and microscopy results. P values lower than 0.05 were considered statistically significant. The statistical program used was STATA (version 10, Stata Corp.).

## Results and Discussion

### Microscopic examination

A total of 519 blood samples were collected from two clinics. Five samples where a clear decision could not be made were removed from further analysis. For the remaining 514 samples, the second reading revealed 12 false negatives, ten samples where mixed species infections were misdiagnosed, and two samples where *P. ovale *was misdiagnosed as *P. vivax*. Thus, for the first reading *vs*. the second reading, the sensitivity was 96.2% (95% CI 94.1%-98.3%); the specificity and positive predictive value were 100% (95% CI in both cases 99.0%-100%), while the negative predictive value was 94.3% (95% CI 91.2%-97.5%). The kappa value calculated to evaluate the agreement on parasite detection was 0.95 (95% CI 0.92-0.98). The kappa value calculated to evaluate species identification was 0.93 (95% CI 0.89-0.97). These levels of concordance are within the normal quality control values, confirming that microscopy reading at the clinics was reliable and accurate.

The microscopy data (parasite species and levels) retained for subsequent comparative analyses were those obtained by the second reading performed by the SMRU microscopists. There were 314 microscopically confirmed *Plasmodium*-infected samples, and 200 samples where no parasites were observed.

### PCR analyses

A total of 413 samples were selected for the PCR analyses. These were 313/314 of the samples for which microscopic examination provided a clear species determination, the last sample was excluded because the amount of blood available was insufficient to conduct all the PCR assays; a random selection of 100 of the 200 samples found to be negative by microscopy were taken for the PCR analysis.

Using this set of samples the primary aims were first to compare the data obtained by two independent laboratories using the same nested PCR protocol, second to compare the influence of the template preparation method on the sensitivity also using nested PCR, and finally to compare the efficacies of nested PCR and multiplex PCR in detecting and identifying parasites. Thus, a total of four data sets were generated (Additional File 1): 1) from the MNHN nested PCR on templates directly purified from blood (Nes-MNHN-B), 2 and 3) from the SMRU nested PCR applied on templates extracted from dried blood on filter paper or directly purified from the blood (Nes-SMRU-B and Nes-SMRU-F, respectively), and 4) from the MSHR multiplex PCR carried out on template extracted from dried blood on filter paper (Mul-MSHR-F). Formal statistical analyses of the results are presented as supplementary data (Additional File 2). The aim of the work presented was to establish the degree of concordance between the data obtained independently in various laboratories. It would have been useful to obtain an exhaustive set of data form each site that would have allowed comparing the performance of all the different template preparation/PCR protocols between laboratories. However, the additional costs and personnel that this would have entailed could not be provided. Ultimately, the work presented was not intended to demonstrate the superiority of one protocol over another, but rather to provide an illustration of the type and extent of variability that might result when different protocols are used or when the same one is used in different settings.

The sensitivity of PCR assays is such that an "all-or-none" result could be obtained when the amount of target DNA in the template aliquot used in the reaction is at the limit of detection. This is especially noticeable for nested PCR, where a few copies of the target gene (from < 10 genomes) usually lead to a positive amplification. Thus, for a sample that contains < 10 parasite genomes per μl of template, when aliquots of one microlitre of template are used in separate assays, there is a probability that sufficient numbers of the target genes are picked up (leading to a positive result) or are not picked up (leading to a negative result) in a particular aliquot. For investigations where detection of very low infections is of paramount importance, samples found negative in a first round of assays are usually subjected to one or more duplicate assays. For the purposes of the present study, it was considered that duplicate assays on a subset of the samples would introduce bias. Thus, the comparisons presented below are based on data obtained from a single PCR assay per sample. It should be noted that for all the PCR datasets, numerous negative controls were included throughout the analyses, and they were invariably negative. The discordances noted between the datasets were therefore, unlikely to be due to contamination.

### Nested PCR assays

In a first instance, templates directly purified from blood were analysed using the same nested PCR assay either at MNHN or at SMRU (Nes-MNHN-B and Nes-SMRU-B), and the data obtained were compared. Of the 313 microscopically positive samples, 312 were also found positive at MNHN, while only 304 positive samples were identified at SMRU. All the microscopically positive samples that were missed by PCR (MNHN or SMRU) had low-level parasitaemias (range 576 P/μl - 16 P/μl of blood). When the parasite species detected in the samples by MNHN and SMRU were compared, discordance was observed in 45 of the 304 positive samples (14.8%). When confronted with the results from the microscopic examination, it became clear that the discordance noted for 44 of these 45 samples was due to a failure to amplify the species present at very low levels alone or as a mixed infection (< 500 P/μl of blood). Failure to detect these parasites was predominantly noted for the SMRU data (42/44), with a species missed in three samples at MNHN (in one case of a very low-level mixed species infection, MNHN missed one species, while SMRU missed the other). The remaining case, where the assay conducted at the SMRU failed to detect a substantial *P. falciparum *parasitaemia (52,501 P/μl of blood), was considered to represent the only true discordance. These results strongly suggested that the sensitivity of the nested PCR assay conducted at the SMRU did not equal that of the same assay conducted at the MNHN. This was confirmed when the nested PCR datasets for the 100 microscopically negative samples were compared. At the MNHN 27 of these samples were found to be *Plasmodium *positive, whereas only five were similarly found positive by the SMRU.

The reduced sensitivity of the nested PCR assay at the SMRU was also reflected in the results obtained using templates purified from filter paper (Nes-SMRU-F). When compared to the MNHN dataset, 302/314 of the microscopically positive samples were identified (312/314 for MNHN), and of these, discordance in species identification between the two data sets was observed for 44 samples. In 38 of these the results from the filter paper nested PCR missed 28 *P. falciparum *and seven *P. vivax *infections, while the assay conducted at the MNHN missed two *P. falciparum *and six *P. vivax *infections. Discordant PCR results were compared to microscopy results, and this revealed that missed and misdiagnosed infections were mainly due to low parasite levels. There were two true discordant results in samples with high levels of *P. falciparum *(1,248 P/μl and 28,637 P/μl blood) missed by SMRU. As for the 100 microscopically negative samples, the assay on templates from filter paper only identified two as positive, thus missing the 25 identified by MNHN.

### Nested PCR assay and template preparation methods

The same nested PCR protocol was applied at the SMRU to templates prepared either directly from blood (Nes-SMRU-B) or from the blood samples dried on filter paper (Nes-SMRU-F). The salient difference between the two template preparation methods resides in the amount of blood that is actually tested in the assay. When prepared directly from blood, the template added to the reaction corresponded to an aliquot of 5.0 μl of blood. Whereas for the template prepared from filter paper, the aliquot added to the assay corresponded to an aliquot of ca. 0.25 μl of blood. Of the 313 microscopically positive samples, the PCR assays failed to detect infection in nine (Nes-SMRU-B) and eleven (Nes-SMRU-F) samples. In all these cases, except for one, the parasitaemia was low (< 576 P/μl of blood). When the parasite species detected at SMRU using the two types of template were compared, discordance was observed for 36 samples, with one species missed in 22 samples for Nes-SMRU-B and in 11 samples for Nes-SMRU-F. For these 33 samples, the species missed was present at low levels (< 176 P/μl blood). The remaining three samples were true discordances, one for Nes-SMRU-B and two for Nes-SMRU-F, in that the assays failed to detect *P. falciparum *parasitaemias of 1248 P/μl, 28637 P/μl or 52501 P/μl of blood, respectively. When the microscopically negative samples were considered, only two were found to be positive by the Nes-SMRU-F assays and only an additional three were detected by the Nes-SMRU-B assays.

### Nested and multiplex PCR assays

Given that the highest sensitivity was obtained by nested PCR assays using the templates directly isolated from whole blood at MNHN, this dataset (Nes-MNHN-B) was considered as the gold standard against which results from the multiplex PCR assays were compared. There were 75 discordant results between the two data sets (Nes-MNHN-B and Mul-MSHR-F). For nine samples, the nested PCR assay missed the detection of one species, while this was the case for 63 samples assayed by multiplex PCR. In all cases the parasite species missed was present at low levels (< 768 P/μl blood). True discordance was only observed in three samples for which the multiplex assay missed two *P. falciparum *(3,840 P/μl or 71,592 P/μl of blood) or one *P. vivax *(7,392 P/μl of blood) infections. Overall multiplex PCR identified 301 of the 313 samples as positive for *Plasmodium*. For the 100 samples that were microscopically negative, multiplex PCR detected infection in 39 samples as compared to 27 samples for nested PCR. *Plasmodium *parasites were detected in these samples by both methods in 12 samples (with one species missed by one or other methods in six of these samples), only by nested PCR in 13 samples and only by multiplex PCR in 25 samples. The apparent higher sensitivity of the multiplex protocol as compared to the nested PCR protocol (39/100 *vs*. 27/100) at detecting parasites in microscopically negative samples might simply be due to chance. Alternatively, it might be due to the DNA breakage that will occur during template preparation, thus favouring the multiplex method where the amplicon sizes for *P. falciparum *and *P. vivax *are 276 bp and 300 bp, as compared to an amplicon size of > 1.6 kb for the primary reaction of the nested PCR protocol. Be that as it may, the multiplex method was clearly inferior at detecting the minor species in samples with mixed species infection, confirming previous observations [8].

### Modification of the epidemiological picture by PCR analyses

The samples analysed in this study were all obtained from symptomatic patients with fever attending a clinic, and as such, the data cannot be approximated to a cross-sectional survey that would be needed to establish the prevalence of malaria parasites in a community/area.

The proportions of the dominant species, *P. falciparum *and *P. vivax*, in the 413 samples was analysed from the data obtained from microscopy and the different PCR assays (Table 1). As compared to microscopy, the nested PCR analyses conducted at the SMRU (Nes-SMRU-B, Nes-SMRU-F) did not in most cases significantly modify the overall proportion of persons infected with *P. vivax *(P = 1.00, P = 0.83, respectively), *P. falciparum *(P = 1.00, P = 0.18, respectively), with both (P = 1.00, P = 1.00, respectively), or none (P = 0.42, P = 0.022, respectively). The multiplex method improved the detection of low level chronic infections missed by microscopy. However, it failed to detect the minor species in many of the mixed infections (*P. vivax *including mixed infections: P = 0.62, *P. falciparum *+*P. vivax *or *P. falciparum*+*P. ovale*: P = 0.63) that were identified by the nested PCR analysis conducted at the MNHN.

**Table 1:** Species prevalence using microscopy compared to different detection methods, paired analysis

Species	**Micros**. SMRU	Nested PCR						Multiplex PCR MSHR-F	
				
		MNHN-B		SMRU-B		SMRU-F			
	
	N (%)	N (%)	P	N (%)	P	N (%)	P	N (%)	P
*Pf*	164 (40)	215 (52)	0.001	163 (39)	1.000	170 (41)	0.180	193 (47)	0.001
*Pv*	173 (42)	193 (47)	0.001	174 (42)	1.000	175 (42)	0.832	169 (41)	0.626
Mixed	29 (7)	72 (17)	0.001	29 (7)	1.000	43 (10)	1.000	24 (6)	0.635
Neg	100 (24)	74 (18)	0.001	104 (25)	0.424	109 (26)	0.022	73 (18)	0.001

Note: N = Sample numbers = N; P = statistical significance (Micros. SMRU used as the comparator). *Pf *= *P. falciparum *and *Pv *= *P. vivax *(for both including mixed infections); Mixed = *Pf*+*Pv *or *Pf*+*Po *(*P. ovale*); Neg = negative. Micros. = Microscopic examination; PCR: Polymerase Chain reaction; MNHN: Museum National d'Histoire Naturelle; SMRU: Shoklo Malaria Research Unit; MSHR: Menzies School of Health Research. B: whole blood sample; F: blood spotted on filter paper

Thus, the data from the Nes-MNHN-B analysis indicated that microscopy missed a substantial number of positive samples (17 *P. falciparum*, eight *P. vivax *and two mixed infections with these two species), or the minor species in mixed infections (*P. falciparum *or *P. vivax *respectively in 31 and 14 samples, respectively). Sub-microscopic vivax malaria in patients diagnosed with *P. falciparum *would have been cleared by the artemisinin-based combination therapy recommended to treat falciparum malaria. However, treatment against *P. falciparum *was inadequate or not provided in approximately 12% (n = 48) of the patients attending the clinics and who were actually infected with this species (in Thailand *P. falciparum *is resistant to chloroquine, the recommended first line treatment for vivax malaria). These cases of sub-microscopic *P. falciparum *might represent infections sampled on the day where the bulk of the biomass was sequestered in the deep vasculature, or cases of chronic falciparum malaria kept in check by acquired immunity to the blood stages. This, or any potential clinical impact on the morbidity, could not be established, because follow-up of screened patients was not included in the current study. Nonetheless, these untreated infections are likely to have been maintained for many days or weeks, thereby increasing the potential to transmit *P. falciparum*.

## Conclusions

The superiority of nucleic acid amplification-based protocols over microscopic examination for the detection and identification of the four classic *Plasmodium *parasite species that infect humans has been amply demonstrated in numerous previous studies. Clearly, it is no longer valid to consider microscopy as the Gold Standard. Moreover, protocols that do not improve on, or at the very least equal, microscopy can be considered to be inadequate or poorly carried out.

In the study presented here, all PCR detection protocols were an improvement on microscopic examination. However, the results obtained when the same set of samples was analysed using two PCR protocols highlighted two hitherto undocumented or neglected aspects. First, the actual contribution of any PCR or amplification-based protocol to enhance the epidemiological knowledge of malaria resides principally in its ability to detect very low numbers of parasites, either in low-grade sub-microscopic infections or as a minor species in mixed infections. Second, there was significant variation in the sensitivity to detect such low level infections when the same protocol (Nested PCR using template obtained directly from the blood sample) was independently carried out in two laboratories. This inter-laboratory variation might be due to one or a combination of factors: for e.g. differences in the enzymes or reagents, the type of thermocycler used, and/or subtle variations in the manner in which template preparation was carried out. Although, the main consequence of this variation was a modest reduction in sensitivity, this was sufficient to obviate the ability of the PCR analysis to improve on the overall parasitological data obtained by microscopy. Indeed, the expense of employing PCR for parasite detection is best justified only when the potential to detect sub-microscopic infections is achieved, for example identification of individuals with persistent sub-patent infections in the context of malaria elimination. The study presented here shows that the choice of the actual methodology used could affect this potential, but it also clearly shows that extrinsic factors could have the dominant impact. The only practical way to ensure consistency in detection sensitivities between laboratories, and indeed between methodologies, i.e. to provide adequate standardization, would simply be to provide a set of centrally prepared standard templates. These standards could take the form of genomic DNA prepared from a fixed volume of blood, supplemented with varying amounts of genomic DNA prepared from the different *Plasmodium *species. Given that the sensitivity of many amplification protocols is close to the lowest levels possible (i.e. <10 copies of the target in the aliquot analysed), a standard sample with this minimal amount of parasite material must be provided. Moreover, standards with parasite DNA from mixed species infections where one species is present as a minority (1:10, 1:100 and 1:1,000 for example) will also be needed. Indeed any protocols that fail to detect low-level infections, alone or in mixed infections, are likely to be of limited usefulness. The availability of this set of standard samples will make it possible to compare the efficacy of various PCR protocols carried out under different conditions.

Meaningful comparison of data generated at different times or from different sites will further require standardising the volume of blood from which the template is obtained: the likelihood to detect parasites present at very low levels, either alone or in a mixed infection, decreases as the volume of blood analysed decreases. Thus, for the detection of sub-microscopic infections or cryptic mixed infections, the DNA templates to be analysed by PCR should correspond to at least 5.0 μl of the whole blood collected from the patient. A PCR analysis of template aliquots corresponding to blood volumes below 0.5 μl will only detect infections that are marginally below the sensitivity of microscopic examination of thick smear. The use of templates isolated from substantially higher volumes of blood (>50 μl) might encounter difficulties due to the high quantities of human DNA (> 1 μg, assuming 8,000 WBC per μl of blood).

In conclusion, molecular detection methods of *Plasmodium *have an important role to play in efforts to control, eliminate and eventually eradicate malaria. There is likely to be a debate on the strategies to deploy these methods, but it is clear that their contribution to the fight against malaria is likely to be valuable only when high detection sensitivities are achieved.

## Competing interests

The authors declare that they have no competing interests.

## Authors' contributions

SP, RP, FN and GS conceived the study. SP, RS, MB, ML, BR and GS designed the experiments. SP, RS, MB, ML, LK, NL, GS performed the experiments. SP, JZ, GS analysed the data. GS wrote the manuscript, with contributions from SP, RS, JZ, RP, ML, BR, and FN. All authors read and approved the final manuscript.

## Supplementary Material

Additional file 1 **Tabulated results of the PCR assays conducted in all sites and for all samples**. The outcomes of all the PCR assays performed on templates prepared using different protocols and conducted in various laboratories, as well as that of the microscopic examination of the samples. Concordance and discordance between templates from different data sets are also presented, as are the resulting overall prevalence data for the different *Plasmodium *species. Click here for file

Additional file 2 **Statistical analysis of the data sets obtained**. Details of the formal statistical analyses conducted to compare the data derived from the various PCR assays/template preparation protocols. Click here for file
