# Supplementary material for: Considerations on the use of nucleic acid-based amplification for malaria parasite detection
Source: Malar J. 2011 Oct 28;10:323. doi: 10.1186/1475-2875-10-323 (PMC3219859; doi:10.1186/1475-2875-10-323)
Supplement: Additional file 2 — Statistical analysis of the data sets obtained. Details of the formal statistical analyses conducted to compare the data derived from the various PCR assays/template preparation protocols. [file 1475-2875-10-323-S2.DOC]

# Additional File 2

Abbreviations: *P. falciparum* = *Pf*; *P. malariae* = *Pm*; *P. ovale* = *Po*; *P. vivax* = *Pv*.

## Number of Plasmodium missed by microscopy compared to PCR methods

The total number of *P. falciparum* missed by microscopy compared to:

- Nes-SMRU-B was 5 (3 *Pf* only and 2 *Pf* in mixed infection)

- Nes-SMRU-F was 8 (1 *Pf* only and 7 *Pf* in mixed infection)

- Nes-MNHN-B was 52 (18 *Pf* only and 32 *Pf* in mixed infection, and 2 mixed infections)

- Mul-MSHR-F was 38 (29 *Pf* only and 7 *Pf* in mixed infection, and 2 mixed infections)

**Table 1: Number of *Plasmodium* missed by microscopy compared to PCR methods**

| *Plasmodium* species | Nes-SMRU-B | | Nes-SMRU-F | | Nes-MNHN-B | | Mul-MSHR-F | |
| --- | --- | --- | --- | --- | --- | --- | --- | --- |
|  | N | Distribution | N | Distribution | N | Distribution | N | Distribution |
| *Pf* (only) | 3 | 18% | 1 | 5% | 18 | 24% | 29 | 55% |
| *Pf* in a mixed infection | 2 | 12% | 7 | 32% | 32 | 43% | 7 | 13% |
| Mixed infection |  | 0% |  | 0% | 2 | 3% | 2 | 4% |
| *Pv* (only) | 4 | 24% | 1 | 5% | 8 | 11% | 12 | 23% |
| *Pv* in a mixed infection | 8 | 47% | 13 | 59% | 14 | 19% | 3 | 6% |
| Total | 17 | 100% | 22 | 100% | 74 | 100% | 53 | 100% |

## Comparison between SMRU and MSHR for filter paper filter spots

The proportion of discordant results between Nes-SMRU-F and Mul-MSHR-F was 23.2% (96/413).

The proportions found positive:

- for *Pf* (including mixed): 41.2% (170/413) in Nes-SMRU-F and 46.5% (192/413) with Mul-MSHR-F

- for *Pv* (including mixed): 42.4% (175/413) and 40.9% (169/413), respectively

- for any mixed infection: 10.4% (43/413) and 5.8% (24/413), respectively

The measure of agreement between these two methods evaluated by the kappa value was 0.74 (95%CI 0.67-0.80), which is interpreted as a good agreement.

Table 1: Comparison of results from Nes-SMRU-F and Mul-MSHR-F

| Nes-SMRU-F | Mul-MSHR-F | | | | | | Total |
| --- | --- | --- | --- | --- | --- | --- | --- |
|  | Negative | *Pf* | *Pf+Po* | *Pf+Pv* | *Pm* | *Pv* |
| Negative | 65 | 26 | 1 | 2 |  | 15 | 109 |
| *Pf* | 5 | 119 |  | 2 |  | 1 | 127 |
| *Pf+Pv* | 1 | 22 |  | 11 |  | 9 | 43 |
| *Pm* |  |  |  |  | 1 |  | 1 |
| *Po* |  | 1 |  |  |  |  | 1 |
| *Pv* | 2 | 1 |  | 8 |  | 121 | 132 |
| Total | 73 | 169 | 1 | 23 | 1 | 146 | 413 |

Note: the kappa value of agreement between two methods is not related to a “gold standard” method. Its value is the same whether one or the other method is regarded as the gold standard.

## Comparison between PCR methods

Table 3: Detection distribution of *Plasmodium* species using different methods

| Species | Microscopy | | Nes-MNHN-B | | Mul-MSHR-F | | Nes-SMRU-B | | Nes-SMRU-F | |
| --- | --- | --- | --- | --- | --- | --- | --- | --- | --- | --- |
|  | N | % | N | % | N | % | N | % | N | % |
| Negative | 100 | 24 | 74 | 18 | 73 | 18 | 104 | 25 | 109 | 26 |
| *Pf* | 135 | 33 | 143 | 35 | 169 | 41 | 134 | 32 | 127 | 31 |
| *Pf+Po* | 1 | <1 | 1 | <1 | 1 | <1 |  |  |  |  |
| *Pf+Pv* | 28 | 7 | 71 | 17 | 23 | 6 | 29 | 7 | 43 | 10 |
| *Pm* | 1 | <1 | 1 | <1 | 1 | <1 |  |  | 1 | <1 |
| *Po* | 3 | 1 | 1 | <1 |  |  | 1 | <1 | 1 | <1 |
| *Pv* | 145 | 35 | 122 | 30 | 146 | 35 | 145 | 35 | 132 | 32 |
| Total | 413 | 100 | 413 | 100 | 413 | 100 | 413 | 100 | 413 | 100 |

Table 2: Discordance comparison between PCR detection methods

| PCR methods compared | Discordance | | | |
| --- | --- | --- | --- | --- |
|  | n | % | 95%CI lower | 95%CI upper |
| Nes-MNHN-B *vs* Nes-SMRU-B | 76/413 | 18.4 | 14.7 | 22.1 |
| Nes-MNHN-B vs Nes-SMRU-F | 83/413 | 20.1 | 16.2 | 24.0 |
| Nes-MNHN-B vs Mul-MSHR-F | 119/413 | 28.8 | 24.4 | 33.2 |
| Nes-SMRU-B vs Nes-SMRU-F | 39/413 | 9.4 | 6.6 | 12.3 |

# Annex

The kappa value between:

- Nes-MNHN-B and microscopy was 0.80 (95%CI 0.72-0.87), which is regarded as a good agreement (≥0.80).
- Nes-MNHN-B and Multiplex was 0.58 (95%CI 0.47-0.69), which is regarded as a moderate agreement.
- Nes-MNHN-B and Nes-SMRU-B was 0.79 (95%CI 0.71-0.86), which is regarded as a substantial agreement.
- Mul-MSHR-F and microscopy was 0.63 (95%CI 0.53-0.72), which is regarded as a substantial agreement.

**Table 3: Comparison on the number of samples found positive with each method (in the 413 patients)**

|  | MNHN* - MICRO | | | MNHN* - MULT | | | MNHN* - SMRU | | | MULT* - MICRO | | |
| --- | --- | --- | --- | --- | --- | --- | --- | --- | --- | --- | --- | --- |
|  | % | 95%CI lower | 95%CI upper | % | 95%CI lower | 95%CI upper | % | 95%CI lower | 95%CI upper | % | 95%CI lower | 95%CI upper |
| Sensitivity | 92.0 | 89.2 | 94.9 | 92.6 | 89.8 | 95.4 | 91.2 | 88.1 | 94.2 | 88.5 | 85.1 | 91.9 |
| Specificity | 98.6 | 96.0 | 100.0 | 64.9 | 54.0 | 75.7 | 100.0 | 99.1 | 100.0 | 83.6 | 75.1 | 92.1 |
| Observed prevalence | 75.8 |  |  | 82.3 |  |  | 74.8 |  |  | 75.8 |  |  |
| True prevalence | 82.1 |  |  | 82.1 |  |  | 82.1 |  |  | 82.3 |  |  |
| PPV | 99.7 | 99.1 | 100.3 | 92.4 | 89.5 | 95.2 | 100.0 | 99.0 | 100.0 | 96.2 | 94.0 | 98.3 |
| NPV | 73.0 | 64.3 | 81.7 | 65.8 | 54.9 | 76.6 | 71.2 | 62.4 | 79.9 | 61.0 | 51.4 | 70.6 |
| Kappa | 0.80 | 0.72 | 0.87 | 0.58 | 0.47 | 0.69 | 0.79 | 0.71 | 0.86 | 0.63 | 0.53 | 0.72 |

*Gold standard

MNHN: Nes-MNHN-B; MICRO: Microscopy; MULT: Mul-MSHR-F; SMRU: NEs-SMRU-B; PPV: Positive predictive value; NPV: Negative predictive value

**Table 4: Kappa values per species on positive samples**

| Per species | | Kappa | | |
| --- | --- | --- | --- | --- |
|  |  | value | lower 95%CI | upper 95%CI |
| Nes-MNHN-B & Microscopy | | 0.74 | 0.67 | 0.80 |
| Nes-MNHN-B & Mul-MSHR-F | | 0.59 | 0.51 | 0.66 |
| Nes-MNHN-B & Nes-SMRU-B | | 0.77 | 0.71 | 0.83 |
| Mul-MSHR-F & Microscopy | | 0.83 | 0.78 | 0.89 |

## Proportions of discordant results

Nes-MNHN-B *vs* Nes-SMRU-B

The proportion of discordant results between Nes-MNHN-B and Nes-SMRU-B was 18.4% (76/413). Among positive results detected by the microscopy, 53 results were discordant in both PCR methods. The main discordance resulted from Nes-SMRU-B that could not detect mixed *P. vivax* results in mixed infection with *P. falciparum* (57%, 30/53).

Two MNHN results were discordant (1%, 2/313) compared to positive microscopy results. In one case Nes-SMRU-B found *P. vivax* and MNHN found *P. falciparum*. In discordant results two out of two (100%) in MNHN method, and in Nes-SMRU-B 42 out of 49 (86%) were mixed infections.

In negative results, the main discordance was resulting from SMRU that could not detect a *P. falciparum* infection (68%, 15/22). The proportion of *P. falciparum* missed overall (including mixed infections) by SMRU was 68% (51/75, 51=30+1+3+15+2). In two cases, MNHN was discordant: a *P. falciparum* and a *P. vivax* in mixed infections (one found by microscopy, and other one found by Nes-SMRU-B).

Nes-MNHN-B vs. Mul-MSHR-F

The proportion of discordant results between Nes-MNHN-B and Nes-MSHR-B was 28.8% (119/413). Among positive results, 74 results were discordant. The main discordance resulted from Mul-MSHR-F that could not detect mixed *P. falciparum* results in mixed infection with *P. falciparum* (41%, 30/74), and *P. vivax* in mixed infection (31%, 23/74).

Nine MNHN results were discordant (3%, 9/313) and 66 multiplex results (21%, 66/313, P=0.001) compared to positive microscopy results. Of these discordant results, 8 out of 9 (89%) for Nes-MNHN-B method, and 56 out of 66 for Mul-MSHR-F (85%) were mixed infections.

In negative results, 44% (44/100) of the results were discordant between the two PCR methods. The main discordance was resulting from Nes-MNHN-B that could not detect a *P. falciparum* infection (36%, 16/44).

The proportion of *P. falciparum* missed overall (including mixed infections) by MNHN was 28.6% (34/119). The proportion of *P. falciparum* missed overall (including mixed infections) by Mul-MSHR-F was 39.5% (47/119). Two results were found *P. falciparum* by Nes-MNHN-B, and multiplex found *P. vivax*, and Nes-MNHN-B found once *P. vivax* when multiplex found *P. falciparum*. In 14% (14/100) the Nes-MNHN-B method, and in 27% (27/100), the multiplex found positive results (P=0.023).

Nes-MNHN-B vs. Nes-SMRU-F

The proportion of discordant results between Nes-MNHN-B and Nes-SMRU-F PCR methods was 20.1% (83/413). Among positive results found in microscopy, 18.5% (58/313) results were discordant between the two PCR methods. The main discordance between these methods resulted from Nes-SMRU-F that could not detect mixed *P. falciparum* results in mixed infection with *P. vivax* (45%, 26/58). Nes-MNHN-B could not detect *P. vivax* in mixed infections in 21% (12/58) of the discordant results.

In negative results, the discordance was resulting from Nes-SMRU-F that could not detect a *P. falciparum* infection (60%, 15/25). The overall proportion of *P. falciparum* missed by Nes-SMRU-F in the discordant was 70% (58/83, and 8% (7/83) by Nes-MNHN-B.

Nes-SMRU-F vs. Nes-SMRU-B

The proportion of discordant results between Nes-SMRU-B and Nes-SMRU-F PCR methods was 9.4% (39/413). Among positive results found in microscopy, 11.5% (36/313) of the results of the two PCR methods were discordant. The main discordance resulted from Nes-SMRU-B that could not detect mixed *P. vivax* results in mixed infection with *P. falciparum* (50%, 18/36). In negative results, the only discordance was resulting from Nes-SMRU-F that could not detect *P. vivax* infection (3%, 3/100).

## Number of discordant results in positive results from microscopy (N=313)

In positive *P. falciparum* (including mixed with *P. vivax*) detected by microscopy (N=163) (*P. falciparum* only, N=135; mixed *P. falciparum* + *P. vivax*, N= 28)

Nes-MNHN-B (1 *Pf* missed)

Nes-SMRU-B (5 *Pf* missed)

Nes-SMRU-F (4 *Pf* missed)

Mul-MSHR-F (9 *Pf* missed)

Number of PCR missed *P. vivax* mixed infection in positive mixed *P. falciparum* + *P. vivax* detected by microscopy (N=28)

Nes-MNHN-B (3 *Pv* missed in mixed *Pf*+*Pv*)

Nes-SMRU-B (4 *Pf*+*Pv* missed in mixed *Pf*+*Pv*)

Nes-SMRU-F (1 *Pf*+*Pv* missed in mixed *Pf*+*Pv*)

Mul-MSHR-F (11 *Pf*+*Pv* missed in mixed *Pf*+*Pv*)

In positive *P. vivax* (only) detected by microscopy (N=145)

Nes-MNHN-B (1 *Pv* missed)

Nes-SMRU-B (4 *Pv* missed)

Nes-SMRU-F (1 *Pv* missed)

Mul-MSHR-F (6 *Pv* missed)

In positive *P. malariae* (only) detected by microscopy (N=1)

Nes-MNHN-B (0 *Pm* missed)

Nes-SMRU-B (1 *Pm* missed)

Nes-SMRU-F (0 *Pm* missed)

Mul-MSHR-F (0 *Pm* missed)

In positive *P. ovale* (only) detected by microscopy (N=3)

Nes-MNHN-B (1 *Po* missed)

Nes-SMRU-B (2 *Po* missed)

Nes-SMRU-F (2 *Po* missed)

Mul-MSHR-F (2 *Po* missed)

In positive *P. falciparum*+*P. ovale* detected by microscopy (N=1)

Nes-MNHN-B (1 *Po* missed)

Nes-SMRU-B (1 *Po* missed)

Nes-SMRU-F (1 *Po* missed)

Mul-MSHR-F (1 *Po* missed)

**In negative results in microscopy (N=100)**

When Microscopy was negative:

- Nes-MNHN-B method found: 17 *Pf*; 2 mixed *Pf+Pv*; 8 *Pv*
- Nes-SMRU-B method found: 2 *Pf*; 3 *Pv*
- Nes-SMRU-F method found: 2 *Pf*
- Mul-MSHR-F method found: 26 *Pf*; 2 *Pf+Pv*; 11 *Pv*

**Table 7: Proportions of discordant between the different methods**

| PCR methods compared | | | Discordance | | | | | |
| --- | --- | --- | --- | --- | --- | --- | --- | --- |
|  |  |  | N | | | % | 95%CI lower | 95%CI upper |
| Nes-MNHN-B | *vs.* | Microscopy | 80 | / | 413 | 19.4 | 15.6 | 23.2 |
| Nes-MNHN-B | *vs.* | Nes-SMRU-B | 76 | / | 413 | 18.4 | 14.7 | 22.1 |
| Nes-MNHN-B | *vs.* | Nes-SMRU-F | 83 | / | 413 | 20.1 | 16.2 | 24.0 |
| Nes-MNHN-B | *vs.* | nest over | 75 | / | 413 | 18.2 | 14.4 | 21.9 |
| Nes-MNHN-B | *vs.* | Mul-MSHR-F | 119 | / | 413 | 28.8 | 24.4 | 33.2 |
| Microscopy | *vs.* | Mul-MSHR-F | 80 | / | 413 | 19.4 | 15.6 | 23.2 |
| Nes-SMRU-F | *vs.* | Mul-MSHR-F | 96 | / | 413 | 23.2 | 19.2 | 27.3 |
| Nes-SMRU-B | *vs.* | s Nes-SMRU-F | 39 | / | 413 | 9.4 | 6.6 | 12.3 |
| Microscopy | *vs.* | s Nes-SMRU-F | 37 | / | 413 | 9.0 | 6.2 | 11.7 |
| Microscopy | *vs.* | Nes-SMRU-B | 30 | / | 413 | 7.3 | 4.8 | 9.8 |
| Microscopy | *vs.* | nest over | 143 | / | 413 | 34.6 | 30.0 | 39.2 |
| Mul-MSHR-F | *vs.* | nest over | 133 | / | 413 | 32.2 | 27.7 | 36.7 |
| Nes-SMRU-B | *vs.* | nest over | 164 | / | 413 | 39.7 | 35.0 | 44.4 |
| Nes-SMRU-F | *vs.* | nest over | 142 | / | 413 | 34.4 | 29.8 | 39.0 |
| Nes-SMRU-B | *vs.* | Mul-MSHR-F | 82 | / | 413 | 19.9 | 16.0 | 23.7 |
